# Patient-reported outcome claims in European and United States orphan drug approvals

**DOI:** 10.1080/20016689.2018.1542920

**Published:** 2018-11-07

**Authors:** Szymon Jarosławski, Pascal Auquier, Borislav Borissov, Claude Dussart, Mondher Toumi

**Affiliations:** a Public Health Department – Research Unit EA 3279, Aix – Marseille University, Marseille, France; b Prescriptia Ltd., Sofia, Bulgaria; c Faculté de Médecine Laennec, Claude Bernard University, Laboratoire Parcours Santé Systémique EA 4129, Université de Lyon, Lyon, France

**Keywords:** Patient-reported outcomes, quality of life, rare diseases, labelling, EMA, FDA

## Abstract

**Purpose:** We aimed to evaluate the rate of usage and the kind of patient-reported outcome (PRO) claims in orphan drug approvals from the European Medicines Agency (EMA) dated between 1/1/2012 and 31/12/2016 and to compare them to those from the US Food and Drug Administration (FDA).

**Methods:** Orphan drug approval documentation was obtained from the EMA website. PRO-related language was extracted from the Summaries of Product Characteristics (SmPCs). Data were compared to a previously published analysis of the FDA approvals from the same time period.

**Results:** Out of 60 approvals that met the inclusion criteria, 12 products approved by the EMA for 13 (21.7%) orphan indications contained PRO language in the Clinical Studies section of the SmPC. Twelve SmPCs contained PRO instruments based on symptoms, five of which also concerned patient functioning. Eight approvals included PRO claims related to quality of life (QoL) most commonly in cancer treatment.

**Conclusion:** The rate of PRO claims was lower for orphan drugs specifically than for all drug approvals by the EMA. However, in accordance with previous findings, the EMA appeared more inclined to grant PRO claims including health-related QoL than the FDA.

## Introduction

A Summary of Product Characteristics (SmPC) is published for every human medicine that has been granted marketing authorisation by the European Medicines Agency (EMA). This document describes properties and officially approved conditions of a drug use, information for healthcare professionals on how to use the drug safely and effectively, and defines the scope of product marketing. SmPCs correspond to the package inserts (PIs) for medicinal products approved by the US Food and Drug Administration (FDA).

The EMA defines a patient-reported outcome (PRO) as ‘any outcome directly evaluated by the patient and based on the patient’s perception of a disease and its treatment(s)’ [1]. It can be measured in absolute terms (e.g., the severity of a sign, symptom, or state of a disease) or as a change from a previous measure [2]. According to the EMA, a PRO can include both single and multi-dimensional domains such as health status and satisfaction with treatment. Health-related quality of life (HRQoL) is a specific type of the PRO, defined as patient’s subjective perception of the effects of the disease and treatment(s) on daily life, well-being, and psychological, physical and social functioning [3]. Data about PRO concepts are collected using PRO instruments such as questionnaires, leaflets, and documentation that support their use [3].

Orphan drugs are products developed to treat rare medical conditions, generally referred to as ‘orphan diseases’. According to EMA, to qualify for orphan designation, a medicine must meet a number of criteria: ‘it must be intended for the treatment, prevention or diagnosis of a disease that is life-threatening or chronically debilitating; the prevalence of the condition in the EU must not be more than 5 in 10,000 or it must be unlikely that marketing of the medicine would generate sufficient returns to justify the investment needed for its development; no satisfactory method of diagnosis, prevention or treatment of the condition concerned can be authorised, or, if such a method exists, the medicine must be of significant benefit to those affected by the condition’ [4].

In order to accelerate patient access to orphan drugs, less evidence is required for their approval compared with non-orphan products. Therefore, orphan drug manufacturers may lack the incentives to collect additional data, such as PROs, during the drug development process. The EMA issued guidance for the use of HRQoL in the evaluation of medicinal products in July 2005, giving broad recommendations but no methodological requirements for the development, validation and use of PROs [5]. However, the EMA requires a pre-specified PRO endpoint in clinical trial documentation in terms of its relationship to other endpoints and suggests the use of HRQoL in addition to efficacy endpoints for a given disease. Further, the PRO used needs to be validated in the qualitative and quantitative research. The agency suggests specific primary and secondary endpoints for most therapeutic fields which may include PROs. For instance, the EMA has encouraged the development of new PRO tools for rare cancers to guide the use of PRO measures in oncology studies, because the existing ones may not be appropriate or specific enough to measure important outcomes in this population [6]. However, PROs are rarely reported in clinical trials of drugs for rare diseases [7,8]. Only 9% of orphan drugs approved by the FDA between 1/1/2012 and 31/12/2016 contained PRO claims in their PIs [9]. In contrast, two studies by Gnanasakthy, et al. reported that 24% and 16.5% of PIs of all drugs approved by the FDA in 2006–2010 and 2011–2015 respectively, included PRO claims [7,10].

Here, we sought to identify and characterise PRO claims in a comprehensive set of EMA orphan drug approvals and to compare the results to the previously published analysis of FDA approvals granted over the same 5-year period [9].

## Methods

A list of orphan drugs approved by the EMA between 1/1/2012 and 31/12/2016 was obtained from the EMA’s website. Vaccines, imaging-related products, products approved based on non-human pivotal trials, products approved based on biosimilarity studies whose SmPC lacked the Clinical Studies section, and products without an orphan indication in the most recent SmPC were excluded. Data on the drugs’ generic name, marketing approval date, approved indication and therapeutic area were extracted from the SmPCs. Further, the Therapeutic Indications and Clinical Studies sections of the SmPC were screened for the use of a PRO tool., All reported outcomes were analysed to identify other kinds of PRO-related language, with respect to whether the data were reported by patients or health care professionals. The identified PRO claims were classified as measures of symptoms, functioning, HRQoL, global patient rating, and ‘others’. Further information on PRO instruments named in the SmPC, the PRO endpoint status (primary, secondary, tertiary/exploratory), the statistical significance of the PRO results reported (yes/no) were collected. Descriptive statistics were performed using Microsoft Excel 2016.

## Results

In the study period, 56 orphan-designated drugs were approved by the EMA for 63 indications. Three of these indications didn’t meet the inclusion criteria: Granupas® and Xaluprine® didn’t have the Clinical section in the SmPCs, and SomaKit TOC® was an imaging-related product. Most approvals were in oncology (39.7%), endocrinology (14.3%) and respiratory (9.5%) therapeutic areas. In terms of areas represented in the final sample, 24 (40%) approvals were in the field of oncology, followed by 9 (15%) in endocrinology and 6 (10%) in respiratory.

Of the 60 indications, 13 (21.7%) of 12 approved products contained PRO language in the Clinical Studies section of the SmPC (Table 1). No drugs contained PRO claims in the Therapeutic Indications section. Eight SmPCs contained a single PRO; four contained two PROs and one contained three PROs. Among the 13 indications with approved PRO claims, six (46.2%) were in oncology, three (23.1%) in respiratory, two (15.4%) in endocrinology, and single in haematology and neurology.

**Table 1.** Orphan drugs with PRO labelling approved by the EMA.

Generic Name	Marketing Approval Date	Approved Indication	Therapeutic area	No of PRO claims	Symptoms	Functioning	HRQoL	PRO tools named in the SmPC	PRO endpoint status	Statistical significance of PRO results	PRO-related language
ixazomib	11/21/2016	In combination with lenalidomide and dexamethasone for the treatment of adult patients with multiple myeloma who have received at least one prior therapy	Oncology	2	yes	yes	yes	EORTC QLQ-C30 EORTC QLQ-MY20	secondary	HRQoL maintained and no statistically significant difference between study arms.	Quality of life as assessed by global health scores (EORTC QLQ-C30 and MY-20) was maintained during treatment and was similar in both treatment regimens in the Phase 3 study
migalastat	05/26/2016	Long-term treatment of adults and adolescents aged 16 years and older with a confirmed diagnosis of Fabry disease (α-galactosidase A deficiency) who have an amenable mutation	Endocrinology	1	yes	no	no	Gastrointestinal Symptoms Rating Scale	primary	yes	Patient-Reported Outcome – Gastrointestinal Symptoms Rating Scale: In the ERT-naïve trial, analyses of the Gastrointestinal Symptoms Rating Scale demonstrated that treatment with Galafold was associated with statistically significant (p < 0.05) improvements versus placebo from baseline to month 6 in the diarrhoea domain, and in the reflux domain for patients with symptoms at baseline
pitolisant	03/31/2016	Treatment of narcolepsy with or without cataplexy	Neurology	2	yes	no	no	Epworth Sleepiness Scale Weekly cataplexy rate (WCR) recorded in patient diaries	primary	yes	PRO 1: To assess the efficacy of pitolisant on Excessive Daytime Sleepiness (EDS), Epworth Sleepiness Scale (ESS) score was used as the primary efficacy criterion PRO 2: Harmony CTP, a supportive double-blind, randomised, parallel group study of pitolisant versus placebo, was designed to establish pitolisant efficacy in patients with high frequency cataplexy in narcolepsy. The primary efficacy endpoint was the change in the average number of cataplexy attacks per week between the 2 weeks of baseline and the 4 weeks of stable treatment period at the end of the study. On the primary efficacy endpoint, Weekly Rate of Cataplexy episodes (WRC), the results with pitolisant were significantly superior to those in the placebo group (p < 0.0001) (The primary endpoint was the change in the average number of cataplexy attacks per week as recorded in patient diaries (weekly cataplexy rate [WCR]) between the 2 weeks of baseline and the 4 weeks of stable dosing period…)
human coagulation factor X	16/03/2016	Treatment and prophylaxis of bleeding episodes, and perioperative management in patients with hereditary factor X deficiency	Haematology	1	yes	no	no	Pre-determined bleed-specific ordinal rating scale	primary	yes	The efficacy of Coagadex in treating bleeding episodes was assessed by the subject and/or investigator for each new bleeding episode, using a pre-determined bleed-specific ordinal rating scale of excellent, good, poor and unassessable.
carfilzomib	11/19/2015	In combination with either lenalidomide and dexamethasone or dexamethasone alone for the treatment of adult patients with multiple myeloma who have received at least one prior therapy	Oncology	1	yes	yes	yes	EORTC QLQ-C30	secondary	yes	Patients treated with KRd reported improved Global Health Status, with higher Global Health Status/Quality of Life (QoL) scores compared with Rd over 18 cycles of treatment (multiplicity unadjusted 1 sided p-value = 0.0001) measured with the EORTC QLQ-C30, an instrument validated in multiple myeloma. The p-values for ORR and Global Health Status/Quality of Life (QoL) scores are descriptive based on the pre-specified multiplicity adjustment plan.
tasimelteon	03/07/2015	Treatment of Non-24-Hour Sleep-Wake Disorder (Non-24) in totally blind adults	Endocrinology	1	yes	no	no	patient-recorded sleep diaries	primary	yes	SET and RESET evaluated the duration and timing of nighttime sleep and daytime naps via patient-recorded diaries.
nintedanib	15/01/2015	Treatment of Idiopathic Pulmonary Fibrosis (IPF)	Respiratory	1	yes	no	yes	Saint George’s Respiratory Questionnaire (SGRQ)	secondary	yes	SGRQ total score measuring health-related quality of life (HRQoL) was analysed at 52 weeks (in both trials INPULSIS-1 and INPULSIS-2)
olaparib	16/12/2014	As monotherapy for maintenance treatment of adult patients with platinum-sensitive relapsed BRCA-mutated (germline and/or somatic) high grade serous epithelial ovarian, fallopian tube, or primary peritoneal cancer who are in response (complete response or partial response) to platinum-based chemotherapy	Oncology	3	yes	no	yes	FOSI (FACT/NCCN Ovarian Symptom Index) Trial Outcome Index (TOI) Functional Analysis of Cancer Therapy–Ovarian total score (FACT-O total)	secondary	no	No statistically significant differences were observed between olaparib and placebo in patient-reported symptoms or HRQoL as measured by improvement and worsening rates in the FACT/NCCN Ovarian Symptom Index (FOSI), Trial Outcome Index (TOI) and Functional Analysis of Cancer Therapy–Ovarian total score (FACT-O total)
obinutuzumab	23/07/2014	In combination with chlorambucil for the treatment of adult patients with previously untreated chronic lymphocytic leukaemia (CLL) and with comorbidities making them unsuitable for full-dose fludarabine-based therapy	Oncology	2	yes	yes	yes	EORTC QLQ-C30 QLQ-CLL-16	secondary	no	In the QLQC30 and QLQ-CLL-16 questionnaires conducted during the treatment period, no substantial difference in any of the subscales was observed
obinutuzumab	23/07/2014	In combination with bendamustine followed by obinutuzumab maintenance for the treatment of patients with follicular lymphoma (FL) who did not respond to or progressed during or up to 6 months after treatment with rituximab or a rituximab-containing regimen	Oncology	2	yes	yes	yes	FACT-Lym EQ-5D	secondary	HRQoL maintained and no statistically significant difference between study arms.	Based on the FACT-Lym questionnaire and EQ-5D index scale collected during the treatment and during follow-up periods, health-related quality of life was generally maintained in the pivotal study with no significant difference between the arms. However, in patients with FL the addition of Gazyvaro to bendamustine delayed the time to worsening of health-related quality of life as measured by the FACT-Lym TOI score by 2.2 months (median 5.6 versus 7.8 months for B and G + B respectively HR = 0.83; 95% CI: 0.60, 1.13)
macitentan	12/20/2013	As monotherapy or in combination for long-term treatment of pulmonary arterial hypertension (PAH) in adult patients of WHO Functional Class (FC) II to III.	Respiratory	1	yes	yes	yes	SF-36	secondary	yes	Macitentan 10 mg improved quality of life assessed by the SF-36 questionnaire (A 36-item, patient-reported survey of patient health)
brentuximab vedotin	10/25/2012	Treatment of adult patients with relapsed or refractory CD30+ Hodgkin lymphoma (HL): 1. following autologous stem cell transplant (ASCT) or 2. following at least two prior therapies when ASCT or multi-agent chemotherapy is not a treatment option. Treatment of adult patients with CD30+ HL at increased risk of relapse or progression following ASCT	Oncology	1	no	no	yes	not specified	tertiary	no	Study SGN35-005 No differences were observed in quality of life between the treatment and placebo arms.
ivacaftor	07/23/2012	Treatment of patients with cystic fibrosis (CF) aged 6 years and older and weighing 25 kg or more who have one of the following gating (class III) mutations in the CFTR gene: G551D, G1244E, G1349D, G178R, G551S, S1251N, S1255P, S549N or S549R; treatment of patients with cystic fibrosis (CF) aged 18 years and older who have an R117H mutation in the CFTR gene.	Respiratory	1	yes	no	no	Cystic Fibrosis Questionnaire Revised (CFQ-R) Respiratory Domain	secondary	no	Mean absolute change from baseline in CFQ-Rb respiratory domain score was evaluated at weeks 24 and 48 CFQ-R: Cystic Fibrosis Questionnaire-Revised is a disease-specific, health-related quality-of-life measure for CF

Abbreviations: ASCT-autologous stem cell transplant; CF-Cystic Fibrosis; CFQ-R-Cystic Fibrosis Questionnaire-Revised; DSSS-Diary Symptom Sum Score; EORTC QLQ-European Organization for Research and Treatment Quality of Life Questionnaire; FACT-Lym- Functional Assessment of Cancer Therapy – Lymphoma; FOSI-FACT/NCCN Ovarian Symptom Index; EQ-5D-EuroQol-5- Dimensional; FACT-O-Functional Analysis of Cancer Therapy–Ovarian; GNDS-Guy’s Neurological Disability Scores; HRQoL-Health-Related Quality of Life; IPF-Idiopathic Pulmonary Fibrosis; MSCS-Mean Symptom Complex Severity; MSCS-Mean Symptom Complex Severity; nOH-neurogenic symptomatic orthostatic hypotension; OHQ-Orthostatic Hypotension Questionnaire; PI-NRS-Pain Intensity Numerical Rating Scale; PI-Package Insert; PRO-Patient Reported Outcome; SF-Short Form; SGRQ-Saint George’s Respiratory Questionnaire; TEQ-Treatment Effect Questionnaire; TOI-Trial Outcome Index; TOS-Treatment Outcome Score; WCR-Weekly cataplexy rate; VAS-Visual Analogue Scale

In seven approvals (53.8% of SmPCs with a PRO claim), the PRO results were statistically significant and in four approvals (30.8%) they were not. In two approvals, PRO results indicated that HRQoL was maintained during treatment versus placebo or an active comparator..

Twelve SmPCs with PRO labels contained instruments that measure disease symptoms, out of which, five also included PRO claims for the patient functioning change, whereas eight approvals included PRO claims for the HRQoL effects.

A PRO was the primary endpoint in four SmPCs (30.8% of approvals with a PRO claim), a secondary endpoint in eight (61.5%) and a tertiary endpoint in one approval. PRO tools that were the primary endpoints assessed disease symptoms exclusively. The most common PRO instrument was the European Organization for Research and Treatment of Cancer Quality of Life Questionnaire (EORTC QLQ-C30), which was quoted in the SmPCs of three oncology products. The remaining instruments occurred only in single approvals.

## Discussion

The EMA approved nearly four times less orphan drugs than the FDA, which granted 195 orphan drug approvals in the same period (Table 2) [9]. This can be partially explained by the fact that the EMA orphan drug regulation was implemented almost 20 years after the US regulation. Additionally, 21.7% of orphan-designated approvals in the studied sample presented PRO claims in their SmPCs. This is significantly below the 47% reported PRO claims for all new drug approvals by the EMA from 2006 to 2010 [11] and the 46% reported from 2008 to 2012 [12]. Conversely to the above mentioned studies, which analysed the entire content of the European Public Assessment Reports (EPARs), we focused only on the SmPCs, because they include PRO claims granted by the EMA. Such a procedure allowed us to avoid including sponsors attempts to achieve such claims. Whereas a decreasing trend in PRO claims was reported for FDA approvals from 2006 to 2015 [7,10], it is unknown if a similar trend occurred at the EMA. However, it is plausible that our PRO rates are still below the more recent PRO claim rates for EMA non-orphan drug approvals; this aspect needs to be confirmed by additional research.

**Table 2.** Comparison of orphan drugs with PRO claims approved by the EMA and FDA between 1/1/2012 and 31/12/2016.

Agency	Number of all orphan drug approvals	Therapeutic areas represented in all orphan drug approvals	Fraction of approvals with PRO claims	Fraction of approvals with PRO claims with statistically insignificant results	Fraction of approvals where PROs were the primary trial outcomes	Three main therapeutic areas represented among approvals with PRO claims
EMA	56	Oncology (39.7%),	21.7%	31%	31%	Oncology (46.2%),
		Endocrinology (14.3%),				Respiratory (23.1%),
		Respiratory (9.5%)				Endocrinology (15.4%)
FDA [9]	195	Oncology (46%),	9%	6%	86%	Haematology (37.5%),
		Haematology, (13.5%),				Neurology (25%),
		Endocrinology (11.8%)				Respiratory (12.5%)

Abbreviations: EMA-European Medicines Agency, FDA-US Food and Drug Administration.

Nevertheless, our study confirms the trend observed for FDA approvals, where orphan drug approvals included significantly fewer PRO claims than the pooled approvals of all drugs. Only 9% of orphan drugs approved by the FDA between 2012 and 2016 had PRO claims in their labels [9] compared to 24% and 16.5% of drugs approved by the FDA in 2006–2010 and 2011–2015, respectively [7,10]. One possible explanation for the low rates of PRO claims for orphan drugs is a low number of dedicated PRO instruments available. Since few PRO tools specific to rare diseases have been developed, the opportunity to achieve a PRO claim on the product label is limited [13].

The higher rate of PRO claims in our study than in the FDA study regarding the same time period [9] could be explained, at least partially, by the fact that the EMA’s guidance on HRQoL was issued in 2005, while the PRO guidance from the FDA was published in 2009. Although both guidance documents were available before our study period, the longer experience of the EMA could have encouraged the industry to seek PRO claims for their products with the agency. Nearly 31% of PRO claims in our sample were based on statistically insignificant results. It would require further research to establish why the EMA was willing to mention these claims in the SmPC rather than omitting them. In comparison, only one out of 16 PRO claims from the FDA approvals were based on results that were statistically insignificant (Table 2) [9].

PROs were the primary trial endpoint for nearly 31% of products with PRO claims, which is slightly below the 37% reported for all EMA approvals in a previous study [12]. However, it is much less than the 71% and 76.7% reported for all new FDA approvals with PRO claims [7,10] and the 86% reported for FDA orphan drug approvals with such claims (Table 2) [9]. This can be partially explained by the fact that, while primary endpoints were typically based on disease symptoms, the FDA-approved PRO claims were symptom-based. Also, the EMA is more inclined to approve HRQoL and functioning measures, although not as primary endpoints [5,11]. All but one approval with a non-primary-endpoint-based PRO claims in our sample involved higher degree outcomes, such as HRQoL or functioning. This suggests that the EMA is more inclined to grant HRQoL-based claims and unlike the FDA, this agency has initially published guidance on the use of HRQoL-based PROs. The FDA’s guidance on the use of all kinds of PRO was limited to oncology products and was published in 2014, so in the second half of the current study period.

However, similar to the FDA-based studies [7,9,10], almost all approvals with PRO claims in our sample featured at least one instrument that measured disease symptoms.

Since the EMA considers HRQoL PROs as supportive evidence only, potentially negative outcomes of these measures should not discourage the industry from collecting them in clinical trials. Being non-primary endpoints, they cannot be used by the agency as a basis to deny drug approval. A lack of deterioration in HRQoL rather than its improvement per se is perceived by the EMA as important information about the drug’s side effect profile. Finally, given the paucity of PRO tools specific to rare diseases, HRQoL can be a useful measure of treatment outcomes from the patients’ perspective.

In terms of therapeutic areas represented in the orphan drug sample, oncology was the main indication in this study, whereas the FDA-based on reported approvals in haematology and oncology were absent [9]. Interestingly, there was only one case of a haematology drug in the current EMA set. The main three therapeutic areas represented in EMA were the same for all orphan drug approvals and the subset of approvals with PRO claims, but only one in haematology was represented in both analogous data sets in FDA approvals (Table 2). This suggests that either the FDA is reluctant to grant PRO claims in oncology and endocrinology orphan drugs or the industry did not pursue such claims. This observation requires further research.

## Conclusions

Orphan drugs have lower rates of PRO claims than all drugs approved by the EMA, but higher rates than orphan drugs approved by the FDA. In line with previous findings, the EMA is also more inclined to grant HRQoL claims than the FDA and to allow such claims for oncology products.
